# Topics of Public Interest

**Published:** 1917-06

**Authors:** 


### TOPICS OF PUBLIC INTEREST

---

**A Learned Profession.** At a recent meeting, physicians quite impartially spoke of diverticuli, diverticulae and diverticula, not to mention that some from a part of the country where the English pronunciation of Latin differs somewhat from the usual standard, pronounced ae to rhyme with lay. All added the Greek ending -itis to the Latin stem, without a qualm. We have also heard of spermatozoae and spermatoboata and one man who sarcastically piled up plurals into spermatazoatae, failed to make an impression. Of course, it is deeds and not words that count, but after all, it is worth while asking whether our influence as a learned profession does not depend somewhat on the avoidance of gross errors. If the use of classic technical terms is not entirely an affectation, it ought to be according to correct standards.

---

**Parturient Mortality.** Recent census statistics give 14.9 deaths directly due to parturition, per 100,000 population. As births are approximately 35: 1000 population, this corresponds to about 4 maternal deaths per 100 confinements. It used to be said that the average parturient mortality was 1%.

---

**Nurses Graduated from the German Deaconess Hospital of Buffalo,** last month: Misses Minnie Fell, C. Irene Griffiths, Bertha L. Clark, Louise J. Clark, Grace Steel, Welhelmina H. Schweitzer, Hazel Esther Hallett, Anna Hewitt, Gertrude A. Grambow. Judging nationality from surnames, one is tempted to ask "What's in a name?"

---

**Modification of Dog Law.** The proposal to allow unlicensed dogs to be shot on sight, based on the danger of sheep killing, is changed to apply only to the country. City dogs remain subject to local ordinances and under control of local humane societies. City dogs visiting the country must wear their tags.

---

**The Robinson Cat License Bill** is to be allowed to die in committee. This is wise from the practical standpoint, however one may regard it in theory. Sometime back in the stone age of the eastern continent, observant human beings learned that the cat was not subject to law and license.

---

**Marriage License Restriction.** The Whitney-Seelye bill requires a verified statement from both parties, that they are free from venereal disease.

---

**Centenarians.** Thomas Edwards, living on a farm near Viola, Wis., was 101 years old April 25. A brother, a few years younger, lives with him. Their parents died at the age of 92. Thomas is a Civil War veteran. Marcus Colf, aged

102, a veteran of the Mexican, Civil and several Indian wars, who has been a resident of the Soldiers' Home at Dayton, O., after trying to enlist for the present war in various cities, was cared for in Buffalo, April 20 by the G. A. R. James Nicholas Vann, who lived in a cabin on a mountain near Middletown, was found dead, April 16. He was said to be 125 years old.

---

**Quack Advertisements** are, by the Marsh bill, made misdemeanors.

---

**Psycho-Analysis Lectures at $5.00** are advertised in a N. Y. paper, and without notice of a benefit for any philanthropy. A good subject for analysis would be the persons who respond.

---

**Standard Oil Earnings.** The New Jersey Co. earned 51½ millions net in 1915 on a capital of 100 millions and a total investment of not quite 150 millions. The Nebraska Co. earned over half a million on a capital of 1 million. The Continental Oil Co. earned 1½ million on a capital of 3 millions. The moral, when for both military and civil purposes, the need of oil products is almost as great as of food stuffs, is plain.

---

**Grain Used for Liquors.** It has been stated that 600,000,000 bushels of grain are used annually for this purpose in this country. Statistics that are said to be more accurate are as follows, in millions of bushels: Beer; corn, 17; rice 2; barley 48. Spirits; corn, 32; rye 3. Total 102 million bushels, equivalent on basis of weight, without regard to differences in nutrient content of different grains, to about 72 million bushels of wheat, and to about 1/7 of the total human consumption of wheat by the population of the U. S. in a year.

---

**Antivaccination Bill.** The Senate Committee has favorably reported the Murphy bill that vaccination shall not be compulsory for attendance at public schools, unless small pox exists in the locality. We favor this bill, simply because practical experience in the present and future, seems to be the only argument that appeals to the antis.

---

**The Golden Rule?** An Ellicottville woman was so anxious to attend a revival meeting that, in spite of the fact that her home was quarantined on account of scarlet fever, she demanded admittance which was refused, as the physician had warned the ushers. She argued that the case of scarlet fever was a mild one, which reminds us of the plea that the baby was such a little one. We also recall the statement made by a minister that the highest religious duty was to save one's own soul.

---

**Railroad Cars Required for Military Purposes.** Lt. Col. Baker of the Q. M. Dept., estimates as follows: For a field army, 80,000 men, including 2 infantry divisions, 1 cavalry division and auxiliary troops 366 trains, aggregating 6229 cars. This is equivalent to 1% of the locomotives, 4.2% of the passenger cars and 0.2% of the freight cars of the whole country. He further specifies as follows: Infantry regiment, 1890 men, 177 animals, 22 vehicles, 85 cars. Cavalry regiment, 54 officers, 1284 men, 1436 animals, 26 vehicles, 150 cars. Artillery regiment, light, 45 officers, 1170 men, 1157 animals, 32 vehicles, 24 guns, 170 cars. Artillery regiment, horse, 45 officers, 1173 men, 1571 animals, 35 vehicles, 24 guns, 194 cars. Engineers, pioneer battalion, 16 officers, 502 men, 165 animals, 12 vehicles, 38 cars. Signal corps, field battalion, 9 officers, 171 men, 206 animals, 15 vehicles, 28 cars.

---

**Fecundity.** A negress, recently arrested in Niagara Falls for disorderly conduct claims to be the mother of 29 children.

---

**State Civil Service Commission Vacancies.** Examinations will be held June 23, application blanks not being sent out after June 11. Assistant Chemist, $1200-$1500; Assistant Medical Inspector of Schools, men only, $3000; Supervising Nurse, Education Dept., $1800. Inspector for State Board of Dental Examiners, $1200. Assistant in Pathology, Institute for Study of Malignant Disease, Buffalo, $1800; Bacteriologist-Pathologist, $2500; Physician Institution for Feeble Minded Children, Syracuse, $1800 and maintenance; Sloyd Instructor, Letchworth Village, $600-$720 and maintenance. Address State Civil Service Commission, Albany.

---

**The Open Air Camp,** conducted by the Buffalo Assn. for the Relief and Control of Tuberculosis, will be opened May 21. Applications for admission should be made to the Central Tuberculosis Dispensary, 175 E. Swan St., or to the Health Centers. Dispensary hours 10-12 daily except Sundays. Telephone, Seneca 1270-M. Hugo A. Brown, Executive Secretary.

---

**Military Potentiality.** It has been stated that Erie Co., N. Y., furnished 30,000 troops for the Civil War, the population in 1860 being 141,971. The population in 1910 was 528,985 and is now undoubtedly at least 5 times what it was at the time of the Civil War. At the same rate, 150,000 troops could be raised from Erie Co. However, in any normal population, the total number of males of military age, even in the broadest sense, is scarcely a quarter of the total and it is usually assumed that only 10% of the population can be utilized for active service. In the Revolution and the Civil War, the total troops amounted to about 11% but, for the first, the service was usually very brief, often amounting only to militia duty during periods of actual danger for the locality. Without being able to deny the statistics of military enlistment for the Civil War, it seems incredible that so large a number could have been raised from the county itself.

---

**Instruction of Venereal Patients.** According to regulations in effect June 1, physicians must furnish every venereal patient with a pamphlet to be prepared by the State Board of Health, containing information to prevent infection of others. Venereal diseases are now recognized by the State authorities as communicable but are not subject to report or quarantine except when the patient is refractory, when power exists to control negligent patients.

---

**Statistics of the Medical Profession.** Polk's Directory for 1916, gives the following numbers of physicians. + signs indicate increases since 1914, = signs indicate that there is exactly the same number. States unmarked show moderate diminution in actual numbers.

| | | | | | | | |
|---|---|---|---|---|---|---|---|
| Ala. | 2490 | Ia. | 3595 | Nev. | 141 | S. D. | 625 |
| Ariz. | 264 | Kas. | 2730 | N. H. | 717+1 | Tenn. | 2909+ |
| Ark. | 2379= | Ky. | 3393 | N. J. | 2789+ | Tex. | 5161 |
| Cal. | 5134+ | La. | 1955 | N. M. | 342 | Utah | 462+ |
| Col. | 1588 | Me. | 1150+ | N. Y. | 14707+ | Vt. | 647 |
| Conn. | 1588+ | Md. | 2177 | N. C. | 1948= | Va. | 2309+ |
| Del. | 271+ | Mass. | 5622+ | N. D. | 583+ | Wash. | 1644+ |
| D. C. | 1077+ | Mich. | 4236+ | O. | 7343+ | W. Va. | 1691+ |
| Fla. | 999 | Minn. | 2353 | Ok. | 2234 | Wis. | 2487 |
| Ga. | 2837 | Miss. | 1683 | Ore. | 1063+ | Wyo. | 164 |
| Idaho | 431 | Mo. | 5946 | Pa. | 10678+ | | |
| Ill. | 10274 | Mont. | 601+ | R. I. | 744+ | | |
| Ind. | 4922 | Neb. | 1898+ | S. C. | 1118+ | | |

The total for the continental U. S. is 134,126 as compared with 132,770 for 1914 and 129,002 for 1912. On the basis of a hundred million population, remembering that fully half of our increase for the last several decades has been due to immigration, which has been practically zero for 3 years, this gives a ratio of 1 physician to 745, the largest for many years. However, this directory is as complete as possible and lists many physicians not in practice. For example, it gives 20,084 for N. Y., N. J. and Conn. while the N. Y. State Directory gives 18,479 for the same area and yet includes many non-practicing physicians but aims to include all that are licensed. On a proportionate basis, there are only 123,408 licensed physicians in the U. S., either practicing or entitled to practice, the ratio being 1: 810, which is still more favorable. (See our issue of Oct. 1914 for previous analyses).

---

**Rockefeller Appropriations.** $475,000 was appropriated at the beginning of the war with Germany for medical research and humanitarian aid. $400,000 additional has been appropriated for the same general purpose. $200,000 has been appropriated for the military work of the Y. M. C. A., toward a fund of $3,000,000. $200,000 has been appropriated for the model Carrel hospital to be established shortly. $60,000 has been appropriated for instructing military and other surgeons in new methods of diagnosis, preparation of serums, etc. $15,000 has been appropriated to provide buildings for a naval psychiatric hospital on the grounds of the N. Y. Marine Hospital.

---

**Eastman Dispensary Dedicated.** The million dollar free dispensary, given by George Eastman to the city of Rochester, was dedicated May 9, during the meeting of the State Dental Society.

---

**Canadian War Casualties.** Up to May 8, the totals are as follows: Killed in action, 774 officers, 14,555 men, total 15,-239. Died of wounds, 242 officers, 5,000 men, total 5,242. Presumed dead, 38 officers, 1,481 men, total 1,519. Died of sickness, 49 officers, 1,151 men, total 1,240. Missing 106 officers, 2,751 men, total 2,857. Wounded but living, condition not stated, 2,743 officers, 60,913 men, total 63,656. It will be noted that the ratio of officers to men in these items is, respectively, 1:19, 1:20, 1:42, 1:23, 1:26, 1:22. For obvious reasons, probably mainly average higher intelligence, the mortality from disease would naturally be less among officers but the difference from the other ratios is less than might be expected, certainly so little as to nullify the charge sometimes made that officers are better protected and better cared for. The slight change of ratio for the missing and the very considerable one for those presumed dead is obviously due to better opportunities for identifying officers. We have no statistics as to the proportion of officers to men but there can be no doubt but that the relative mortality among the former, as well as their exposure to wounds and disease is disproportionately high. The comparison between deaths immediately or subsequently due to wounds and those from sickness is most remarkable, especially when we consider that, except as mitigated by sanitary science, the sanitary and hygienic conditions of the present war have been far worse than those of any other major war for more than 100 years. Less than 1 death occurred from sickness to 16 from wounds. In the Civil War, the deaths from disease outnumbered those from wounds, almost 2:1, and by more than this ratio if deaths from disease in Confederate prisons are included. They amounted to about 4:7 of all deaths while, in the present instance, they are only about 1/20 of the total. The present war is commonly thought of as being unusually bloody but a comparison with the Civil War scarcely bears out this idea. In the latter, almost exactly 4% of the total troops raised by the north were killed in action (including delayed deaths from wounds) in 4 years. In the present war, the deaths of the Canadian troops have been between 4 and 5% in 2¾ years. It is obviously impossible, in either case, to determine the risk to a given unit of troops for a definite unit of time with or without actual combat. In neither case has the total force been continuously in active service and it is doubtful whether even after the close of the war such a hazard can be calculated mathematically. Allowing for the fact that fighting has been almost continuous on the western front and that it was subject to considerable intermissions in the Civil War and that remissions of hostilities due to weather have been considerable greater, relatively, in the present war on account of higher latitude, it may be questioned whether the average combatant mortality differs greatly in contrasting the two wars.

---

**Support for Day Nurseries.** A bill has been passed requiring the common council of Buffalo to appropriate $500 for each day nursery in the city.

---

**Base Hospital Offer.** The Buffalo Municipal Hospital, now under construction, has been tendered to the Government for a base hospital. It will be completed in about six months, will accommodate 500 patients or, by crowding, 750, and has 80 acres of land available for convalescent camps.

---

**Credit Bureau.** The St. Louis Medical Society has opened a collection bureau for the use of its members. Following are its purposes: (1) Confidential ratings on patients by

'phone; (2) Collect current accounts for 10 per cent; four months accounts to one year, 30 per cent; over one year and under two years, 35 per cent; over two years old 50 per cent; all accounts under $5.00, 50 per cent; (3) monthly statement of amount collected with check for the amount, less the cost of collection.—Med. Herald.

---

**Automobile Statistics.** Up to May 1, the automobiles licensed in N. Y. State, not counting licenses to dealers, numbered over 293,000, beside 17,905 motor cycles. The figures by districts are as follows:

| | Buffalo. | New York. | Albany. |
|---|---|---|---|
| Pleasure ............. | 81,480 | 100,584 | 64,463 |
| Omnibus ............ | 1,045 | 3,960 | 2,478 |
| Commercial .......... | 9,171 | 23,354 | 6,672 |
| Dealers ............. | 750 | 867 | 797 |
| Chauffeurs .......... | 13,037 | 68,572 | 13,571 |
| Receipts ............. | $779,006.50 | $1,585,424.50 | $642,515.00 |

The total revenue for this year will be about four million, which should provide for 150-200 miles of brick or cement roads.

---

**The Children's Hospital** of Buffalo has abandoned its proposed campaign for $50,000, on account of the pressing economic conditions. An attempt will be made to raise $30,-000 for immediate needs, by strictly voluntary subscriptions. This action cannot be too highly commended. Forced philanthropy at the beginning of the war can easily exhaust the surplus wealth in the hands of the well disposed, and apply it to activities, well enough in their way but not so vitally important as others whose need will be felt later. An equable, universal, official distribution of the burdens of war and of routine philanthropy is the best method, to obtain either men or money.

---

**Centenarian.** Mrs. Elizabeth Snively died at the Niagara Falls General Hospital, May 6, aged 100.

---

**Hydrophobia.** Claude Wilson of Sugartown Valley near Salamanca died May 1, following a dog bite seven years ago. (Is so long a period of incubation possible? Ed.)

---

**Protection of Physicians Absent on Military Duty.** The State Society has undertaken to inforce the recommendation of the Surgeon General's suggestion to hold together the practice of physicians in military service. Blank forms have been prepared in triplicate for notification of patients as to substitute, of the substitute himself, while the third blank will be held by the Secretary of the State Society. Substitute physicians are to turn over one-third of all fees received and to agree not to attend the patient within a year of the return of the original family physician. This impresses us as a practical and reasonable arrangement but there is a serious obstacle to the carrying out of any such plan—the modern tendency to break away from the old ideal of a definite family physician. However the method automatically provides, so far as the substitute is concerned, for any conflict of this nature and, if the patient sees fit to ignore the reference to a substitute, his right of selection is unimpaired. Specialists, of course, cannot be so well protected as men with family practices and must depend largely on the honor and good will of the profession on their return to private practice.

---

**Poliomyelitis Clinic.** The State Dept. of Health held a clinic in Buffalo, May 9, under the direction of Dr. Lovett of Boston, for patients and physicians of Erie, Niagara, Orleans, Genesee and Chautauqua Cos. 131 patients were examined, the work being continued for three days.

